# Supplementary material for: Limited impact of hydrogen co-firing on prolonging fossil-based power generation under low emissions scenarios
Source: Nat Commun. 2024 Mar 4;15:1778. doi: 10.1038/s41467-024-46101-5 (PMC10912371; doi:10.1038/s41467-024-46101-5)
Supplement: Supplementary file 1 — Supplementary Information [file 41467_2024_46101_MOESM1_ESM.pdf]

Supplementary Information

Limited impact of hydrogen co-firing on prolonging fossil-based power generation under low emissions scenarios

Ken Oshiro [1*]
Shinichiro Fujimori [1,2,3]

1. Kyoto University, C1-3, Kyotodaigaku-Katsura, Nishikyo-ku, Kyoto, Japan
2. National Institute for Environmental Studies, Tsukuba, Japan
3. International Institute for Applied Systems Analysis (IIASA), Laxenburg, Austria

* Corresponding author (ohshiro.ken.6e@kyoto-u.ac.jp)

Supplementary Figures

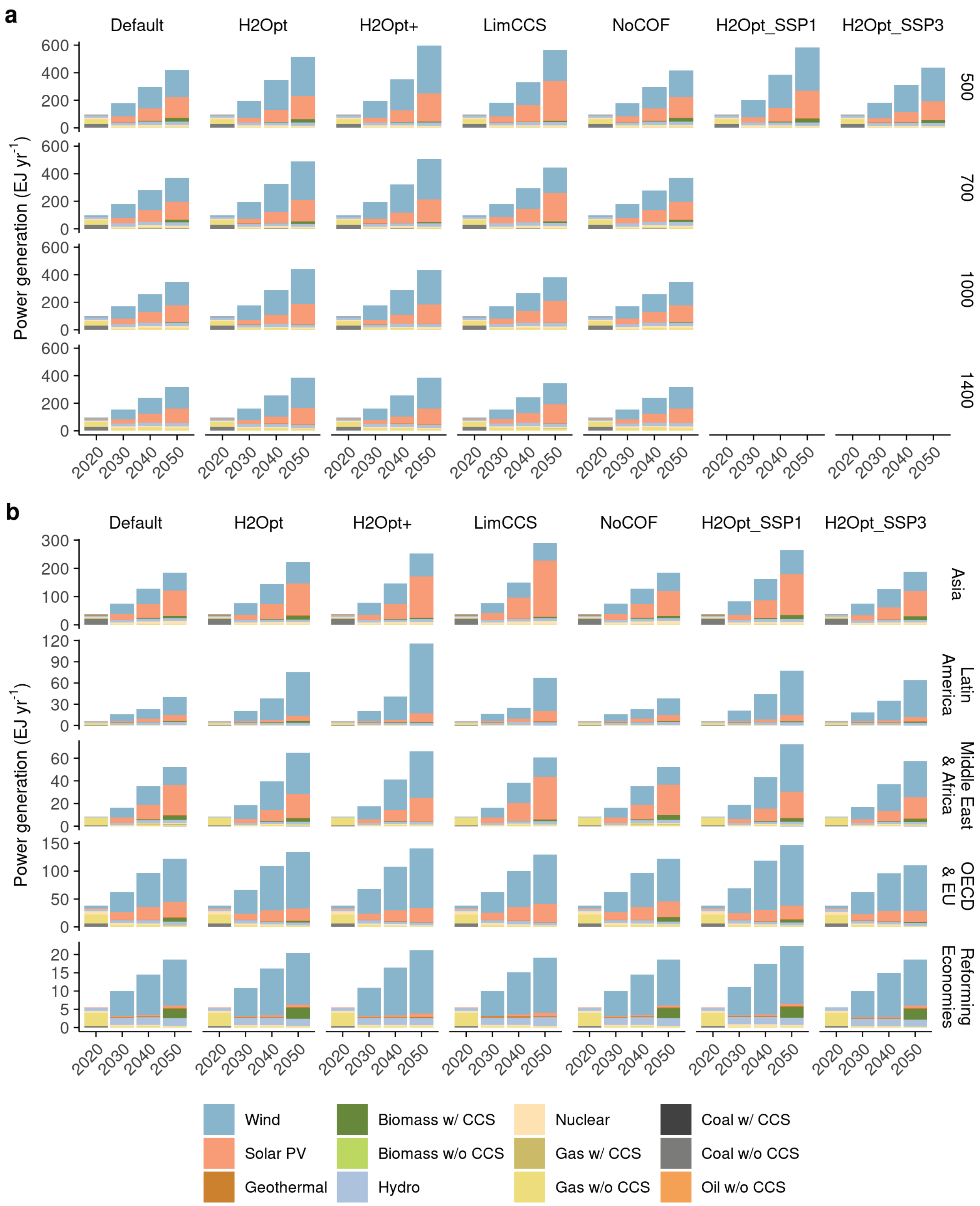

Supplementary Fig. 1. Power generation. a Power generation across all mitigation scenarios. **b** Power generation by region in the 500 Gt-CO_2 budget scenarios.

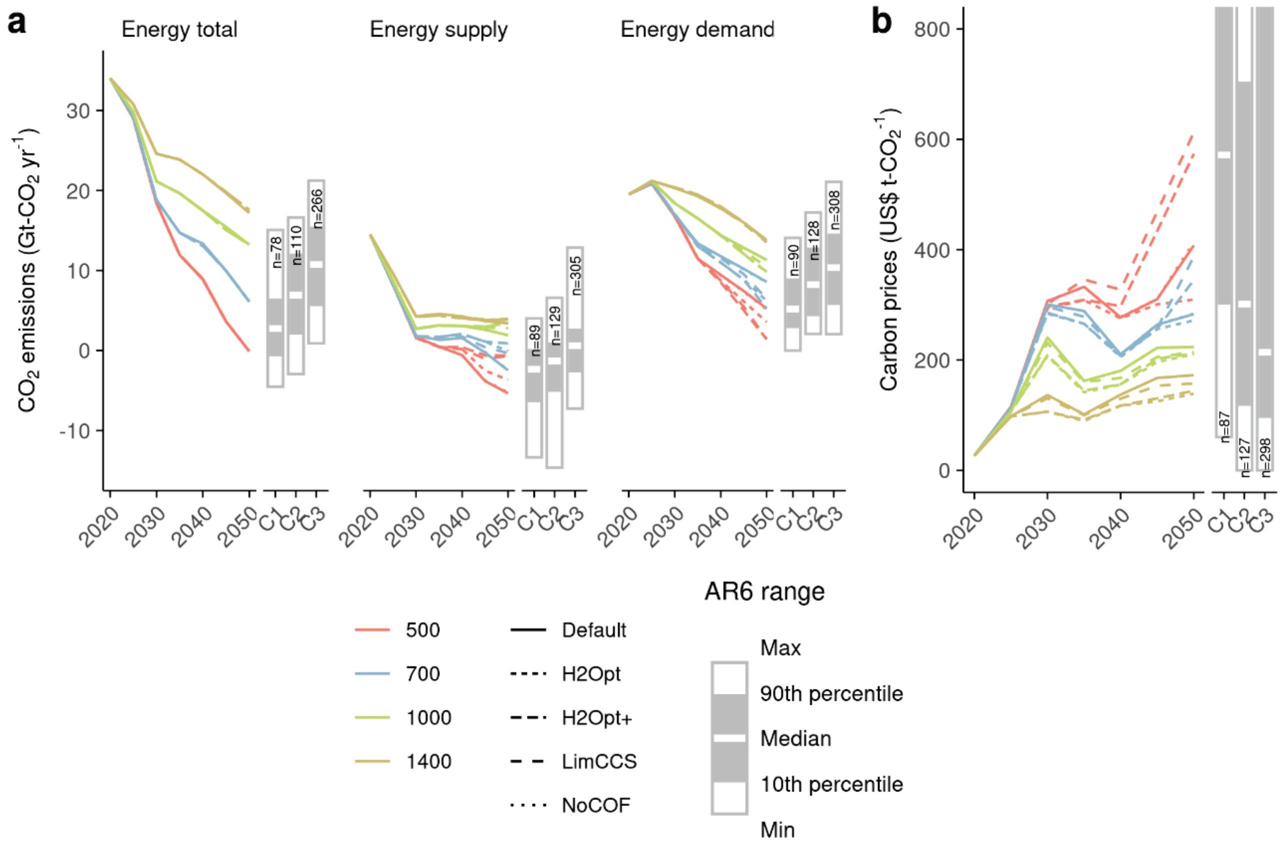

Supplementary Fig. 2. Emissions and carbon prices. a CO_2 emissions from the energy supply and demand sectors. Emission total includes the negative emissions by direct air carbon capture and storage (DACCS). **b** Carbon prices. Right bar plots illustrate the values in 2050 as obtained from the Intergovernmental Panel on Climate Change Sixth Assessment Report (IPCC AR6) [1]. Right bar plots illustrate the capacity of total fossil fuel-fired generators in 2050 in the IPCC-AR6. "n" denotes the number of available scenarios in each category. The range of 0-800 US$ per t-CO_2 is illustrated.

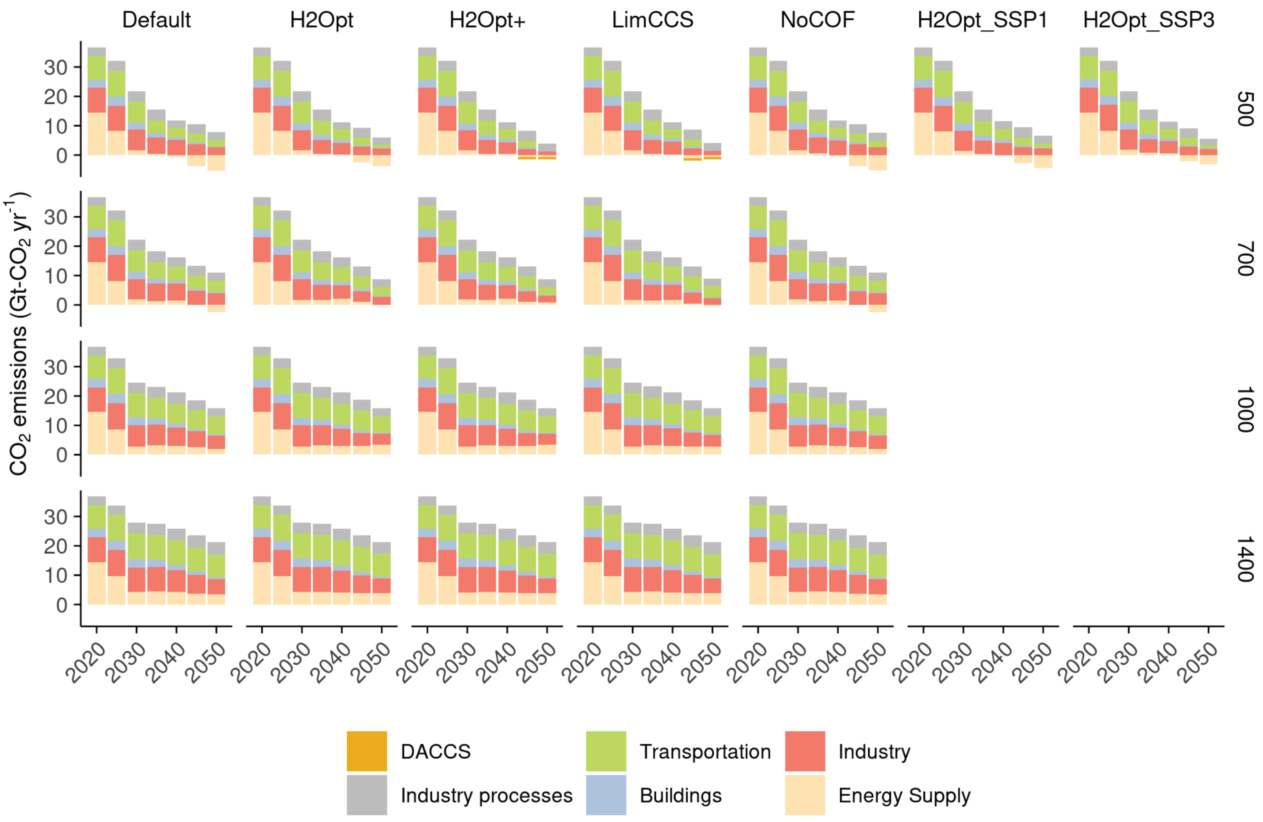

Supplementary Fig. 3. CO_2 emissions by sector.

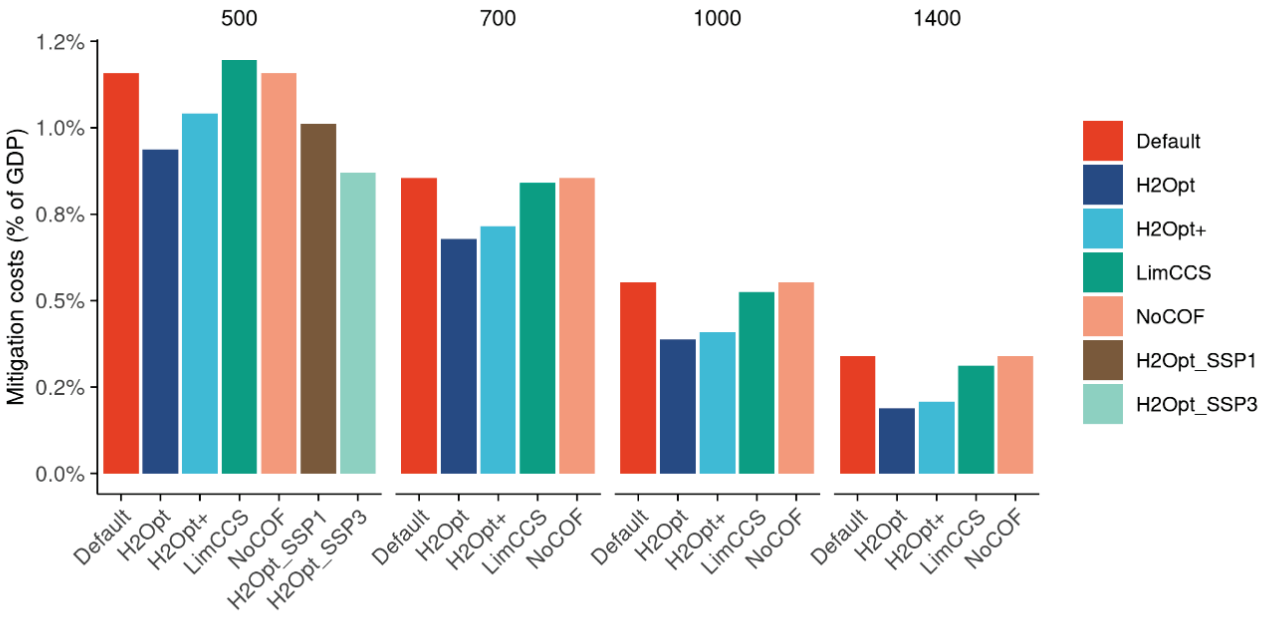

Supplementary Fig. 4. Cumulative additional energy system costs between 2021 and 2050 as a fraction of GDP, discounted by 5% per year.

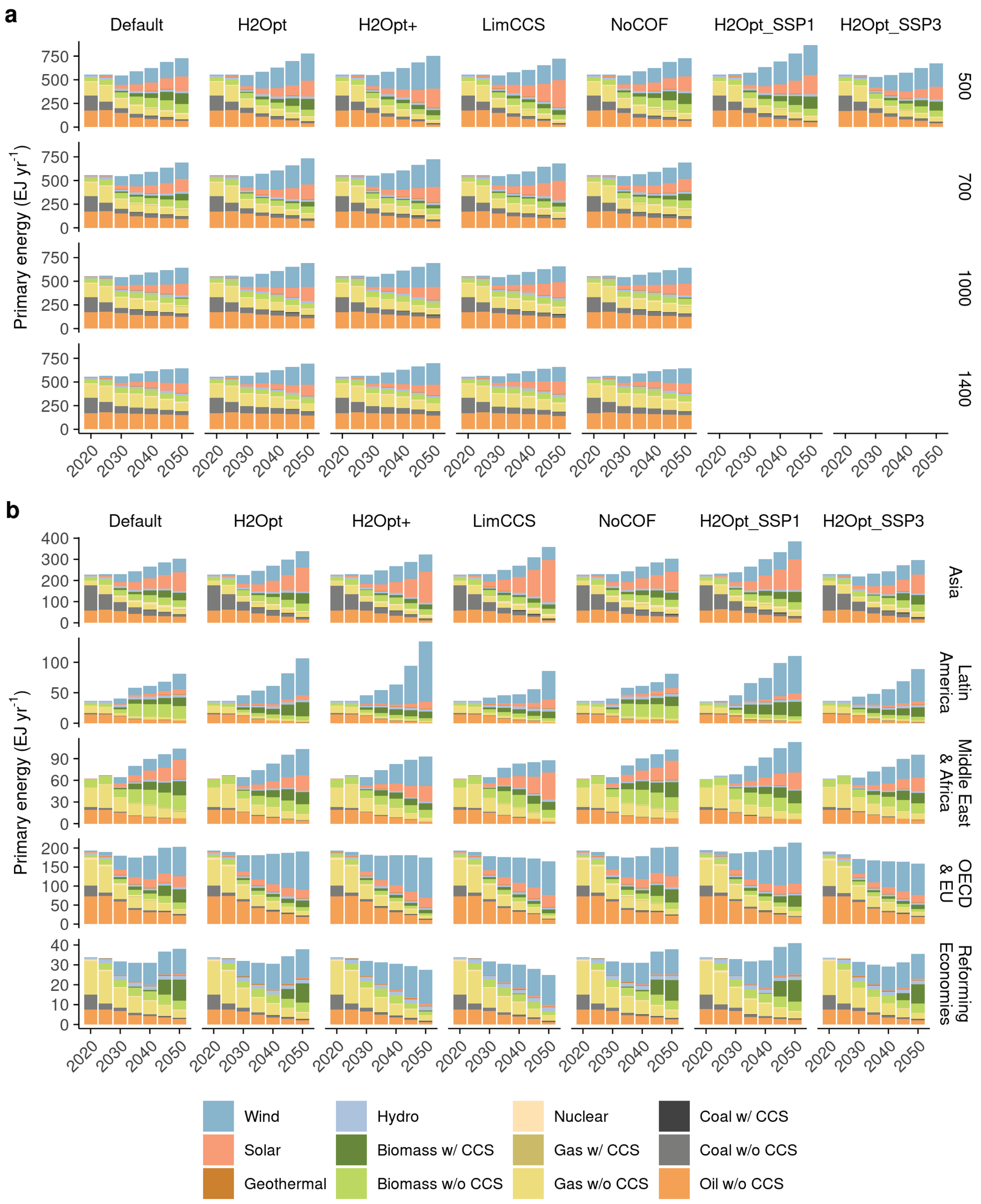

Supplementary Fig. 5. Primary energy supply over time. a Primary energy across all mitigation scenarios. **b** Primary energy by region in the 500 Gt-CO_2 budget scenarios

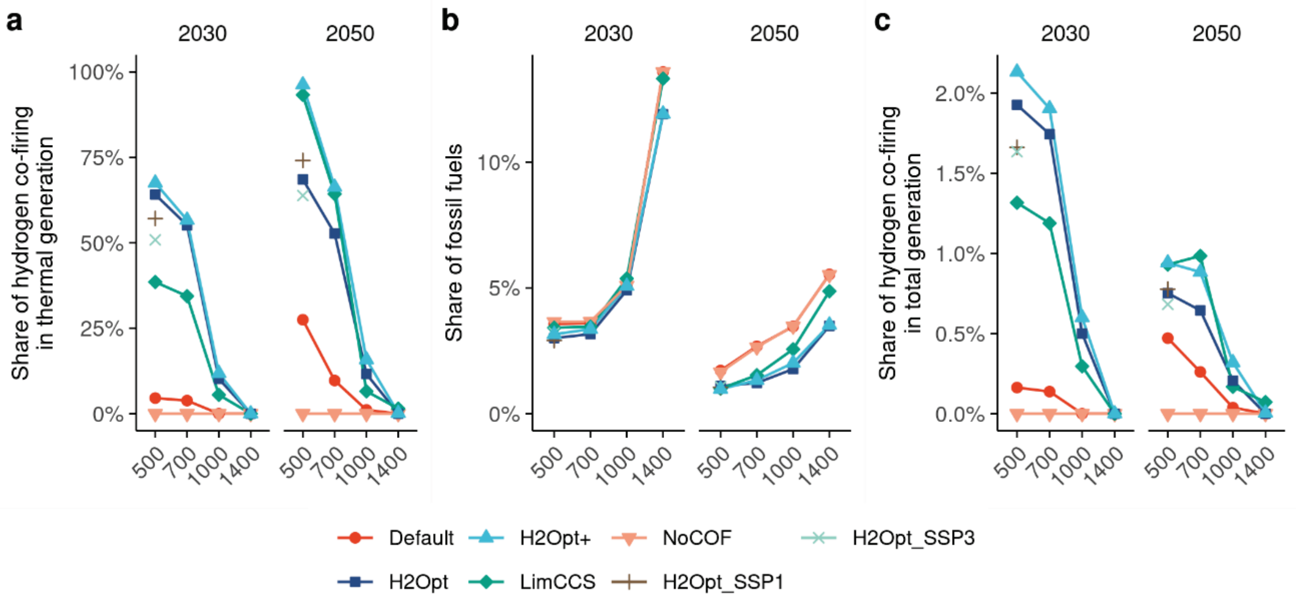

Supplementary Fig. 6. Share of hydrogen co-firing in the power sector, including the sensitivity scenarios. a Hydrogen co-fired power generation as a share of total thermal power generation. **b** Fossil fuel-fired power generation, including hydrogen co-firing, as a share of total power generation. **c** Hydrogen co-fired power generation as a share of total power generation.

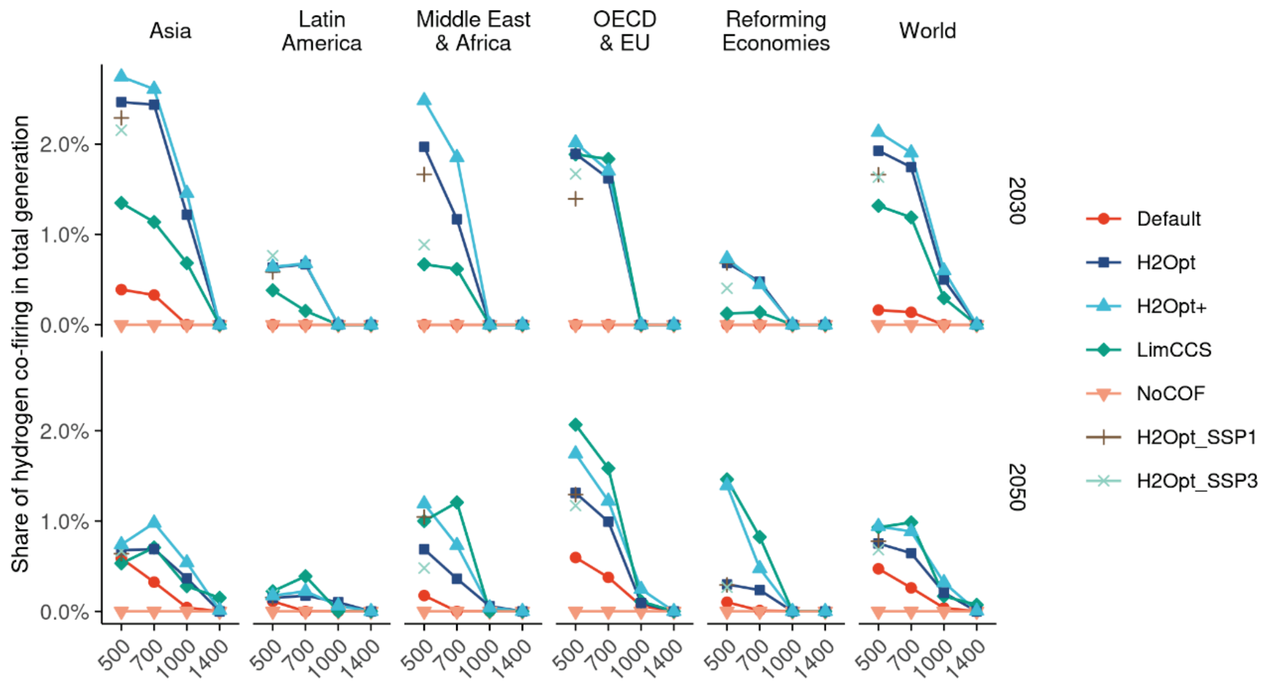

Supplementary Fig. 7. Hydrogen co-fired power generation as a share of total power generation by region.

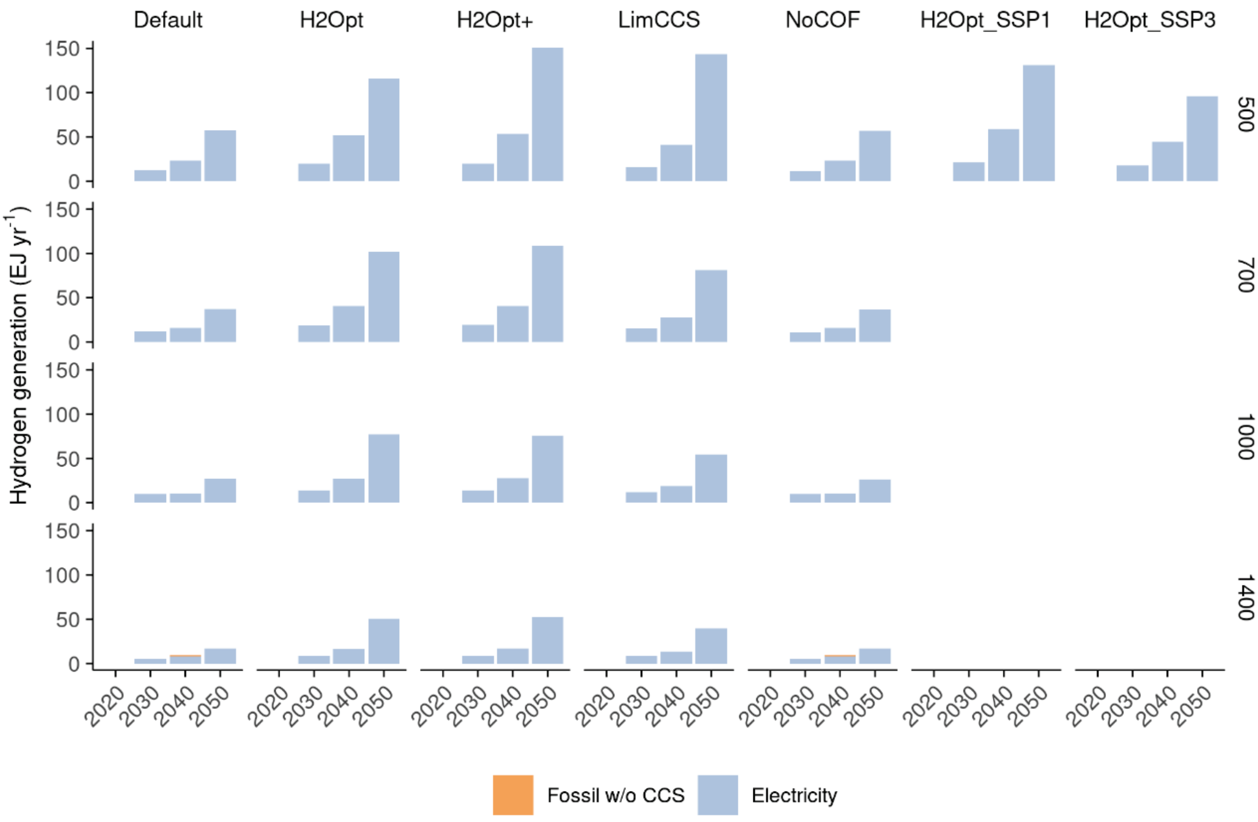

Supplementary Fig. 8. Hydrogen generation by source.

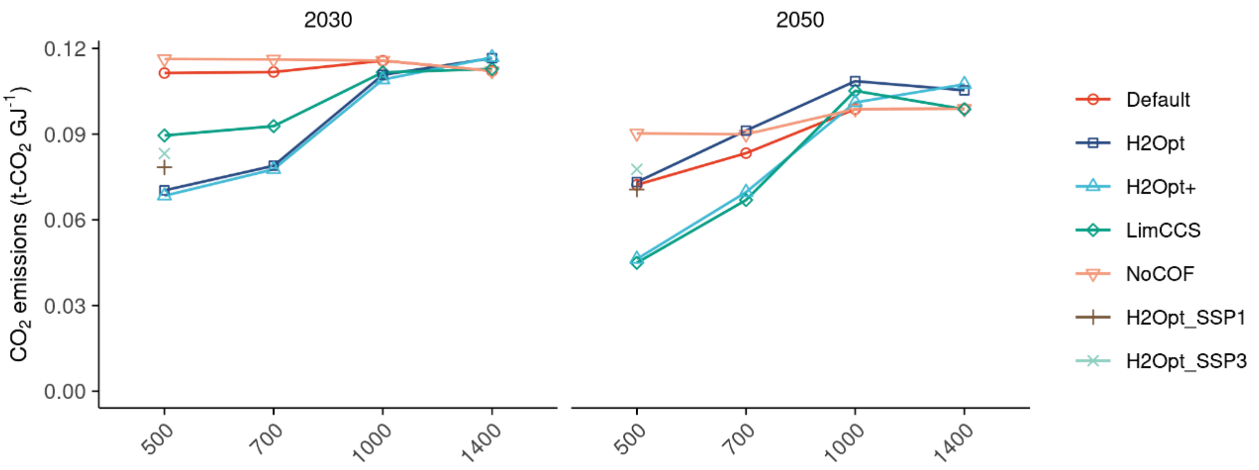

Supplementary Fig. 9. Average emissions intensity of fossil fuel-fired electricity generation.

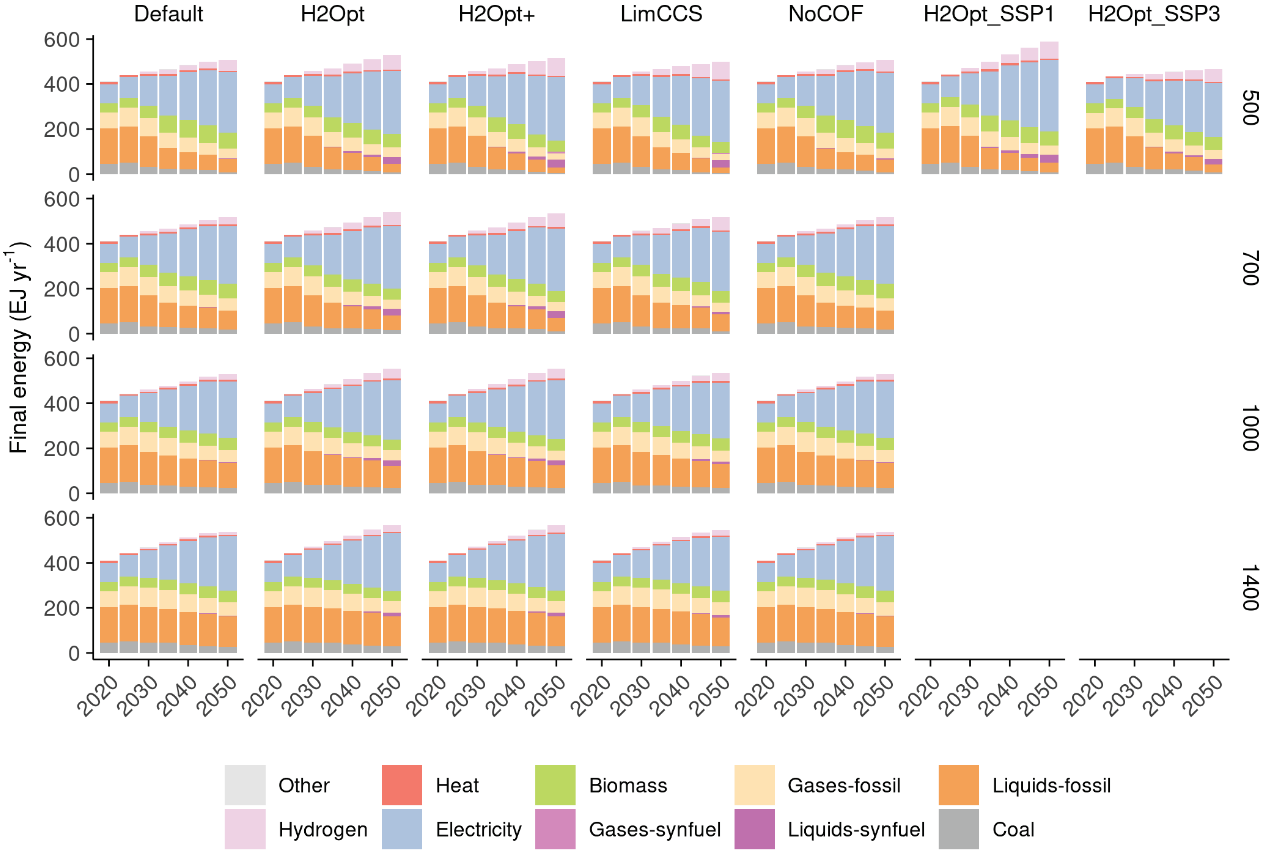

Supplementary Fig. 10. Final energy demand over time.

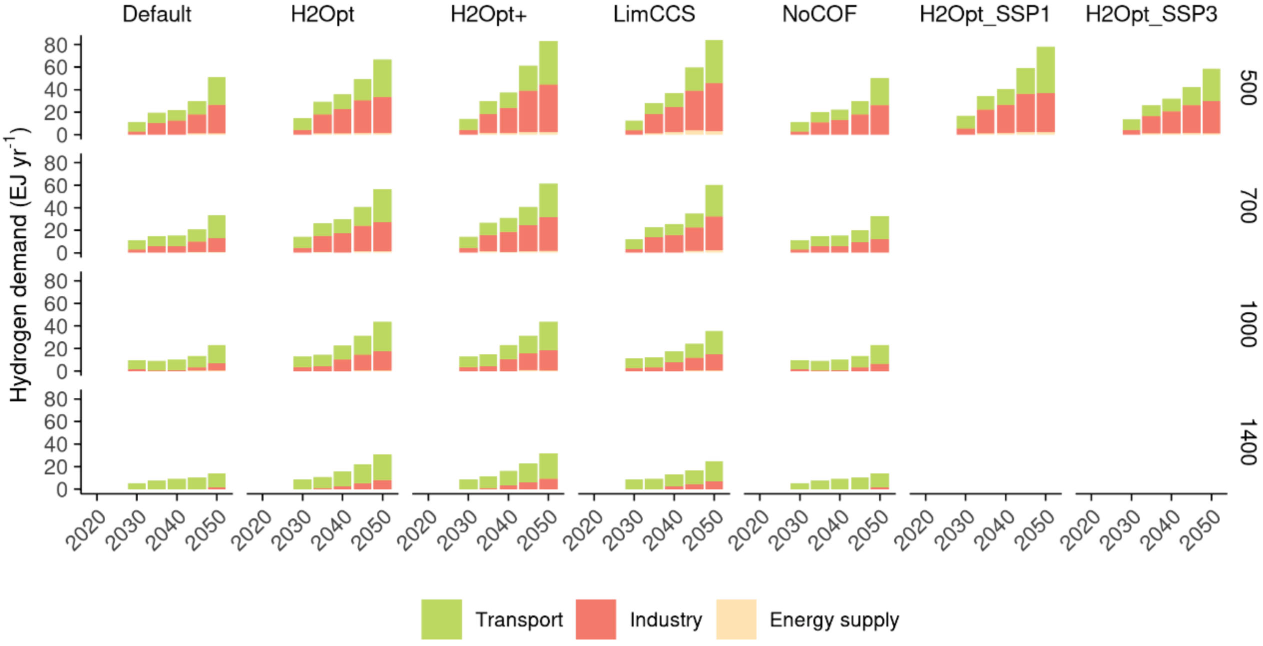

Supplementary Fig. 11. Hydrogen demand in energy sectors.

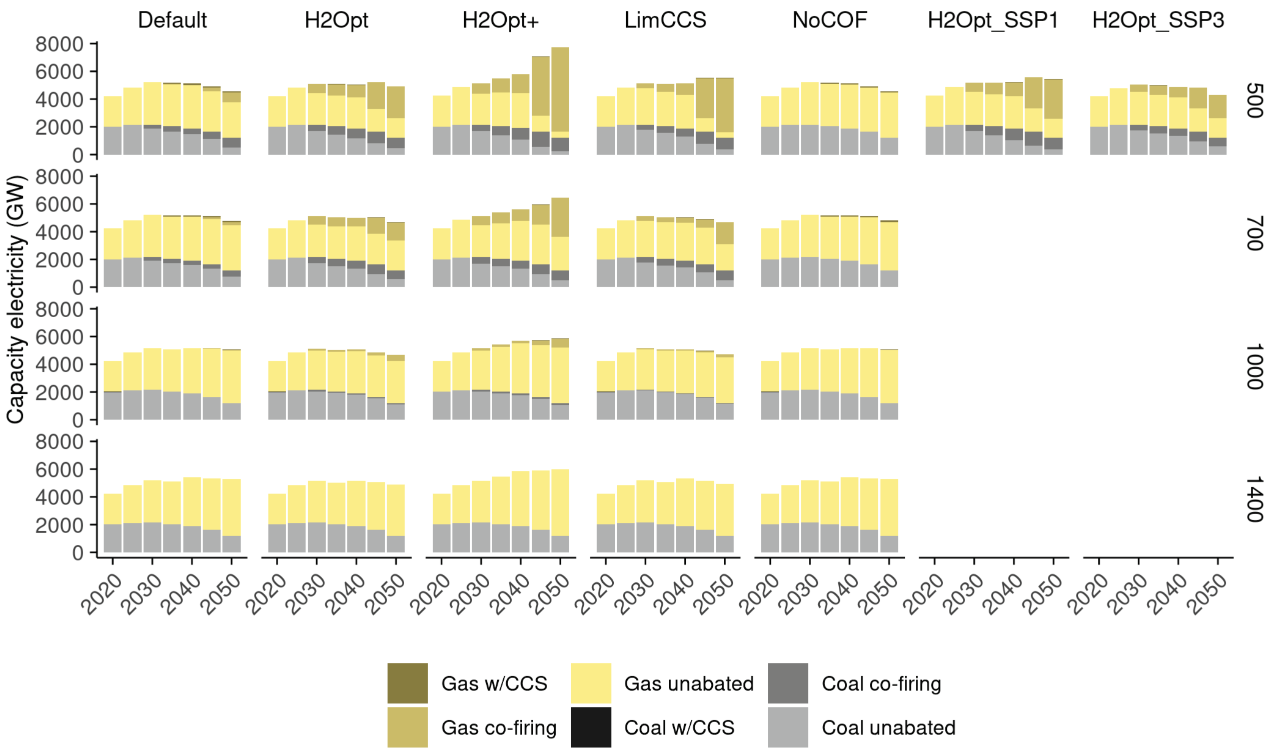

Supplementary Fig. 12. Capacities of fossil fuel- and hydrogen co-fired powerplants over time.

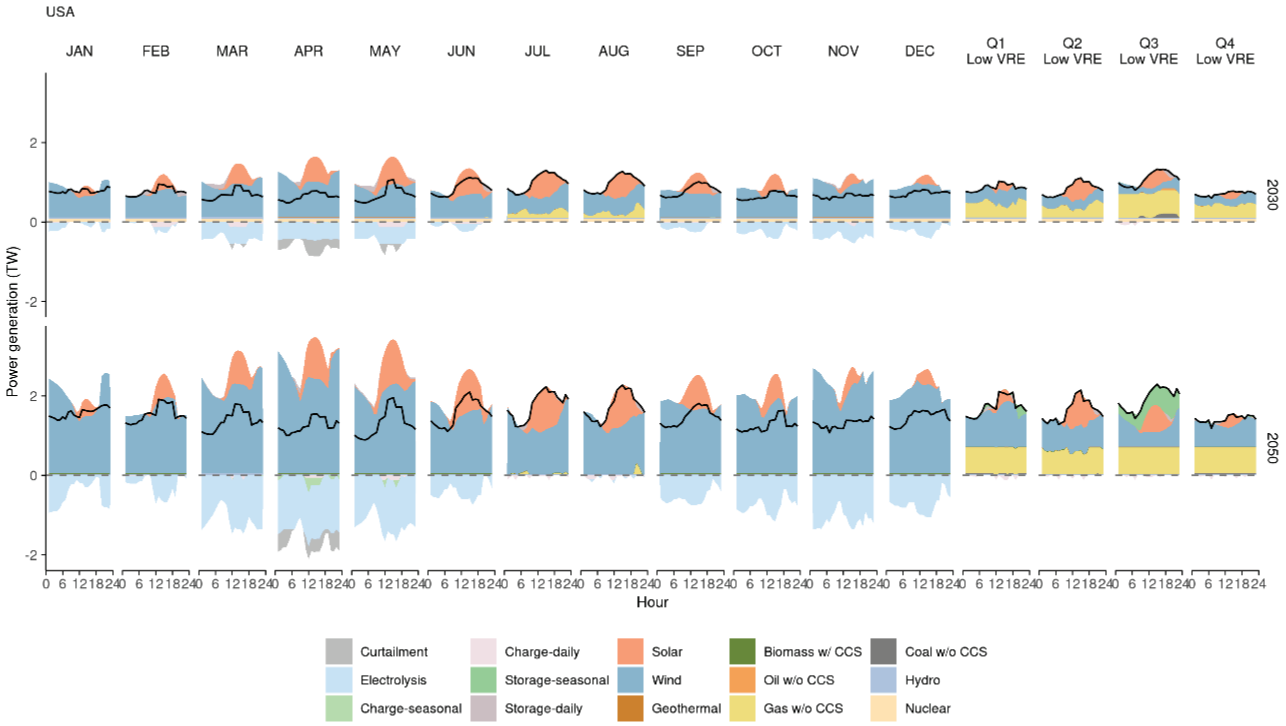

Supplementary Fig. 13. Hourly power generation in the US during modeled representative days under the 500-H2Opt scenario. Solid line represents the hourly electricity demand.

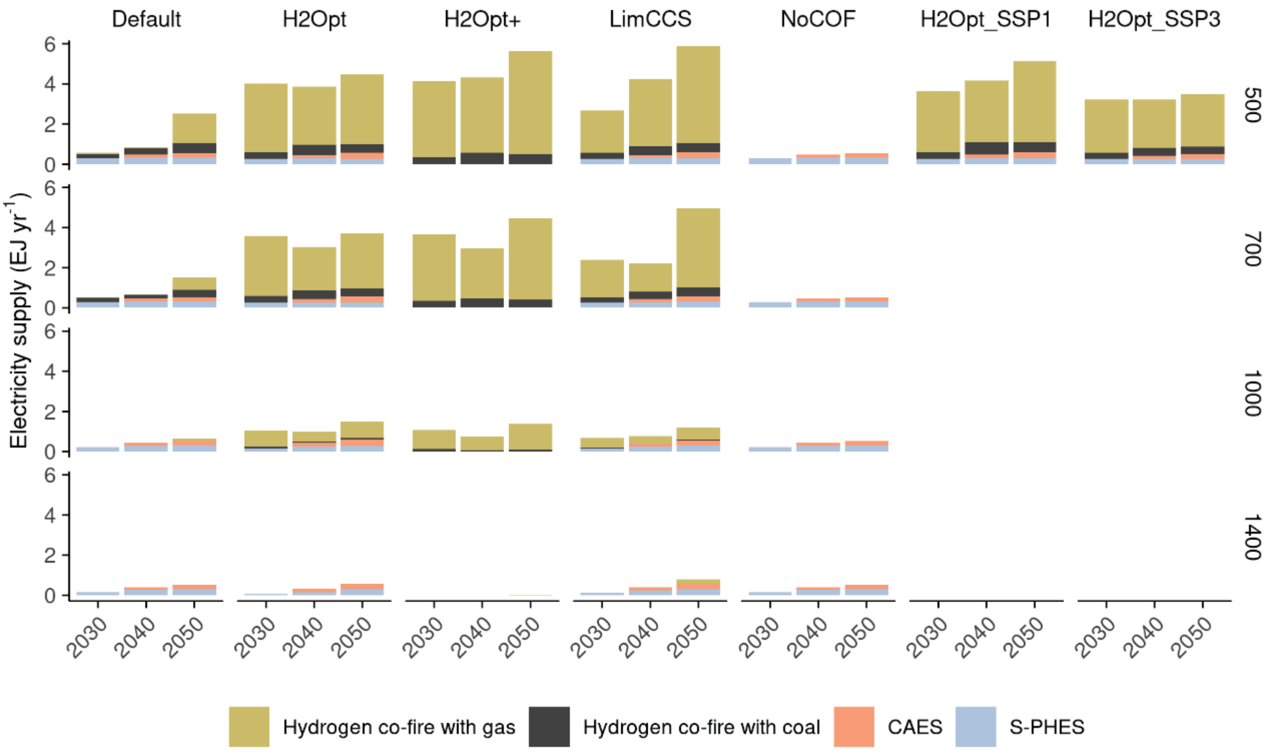

Supplementary Fig. 14. Annual power supply from various seasonal storage options.

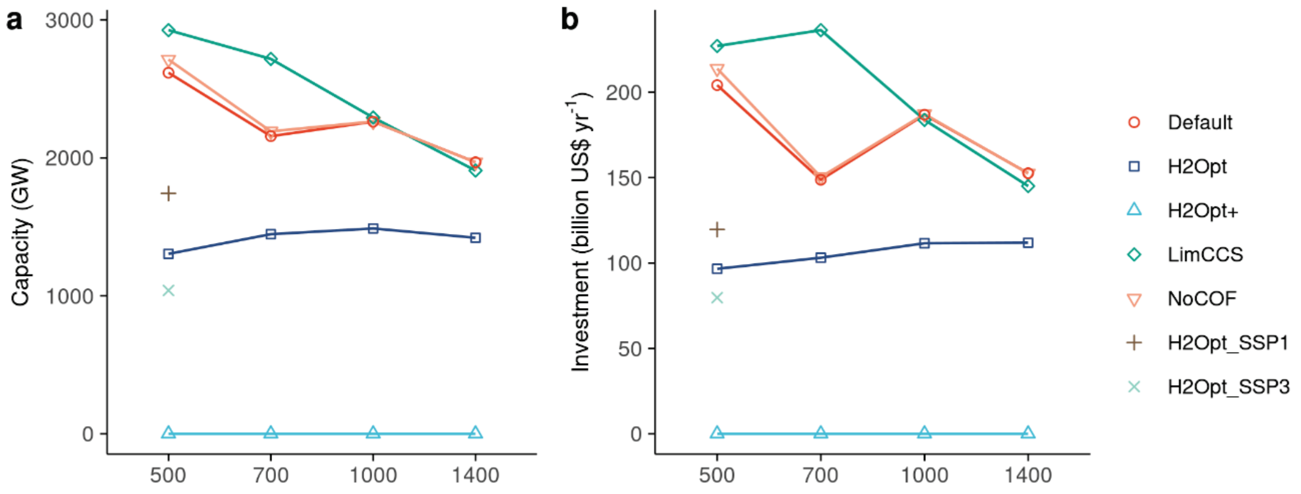

Supplementary Fig. 15. Battery storage. a Battery storage capacity in 2050. **b** Annual investment for battery storage in 2050. The installation of battery storage does not occur in the H2Opt+ scenario because of the high cost assumptions of this scenario.

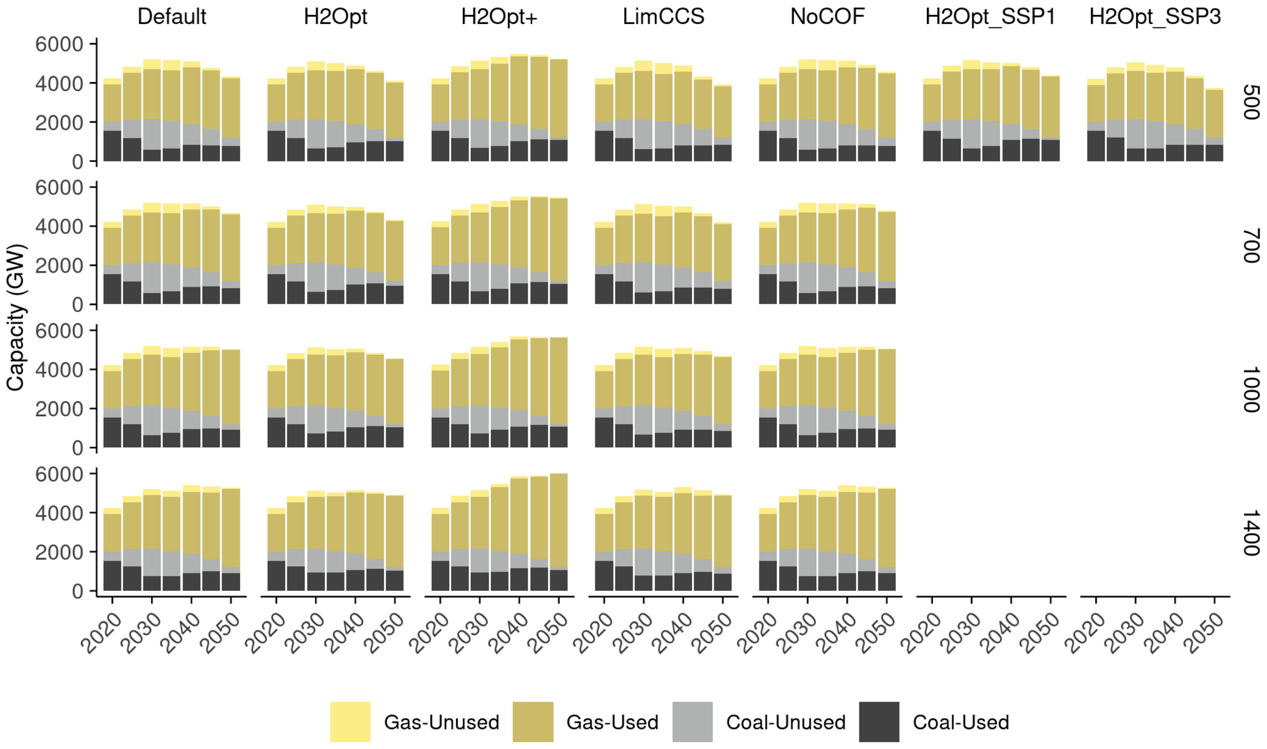

Supplementary Fig. 16. Capacity of fossil fuel-fired generators.

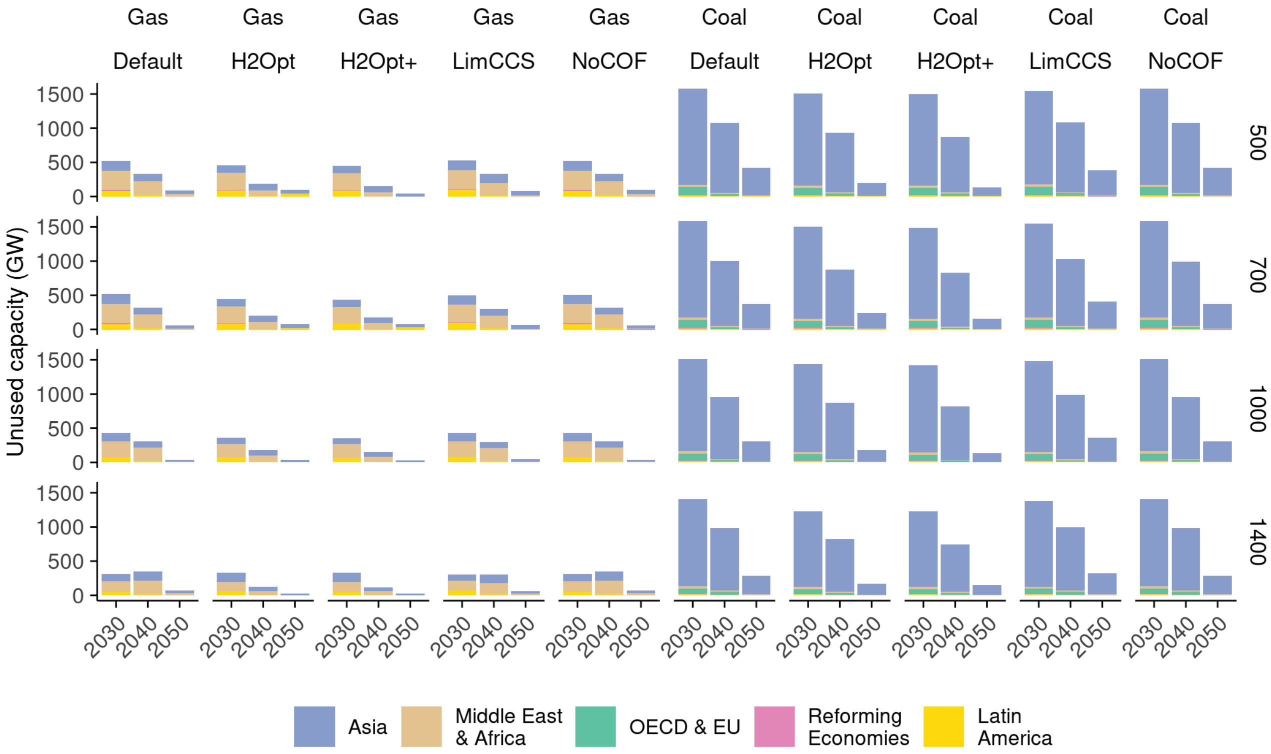

Supplementary Fig. 17. Stranded capacity of coal and gas generators by region.

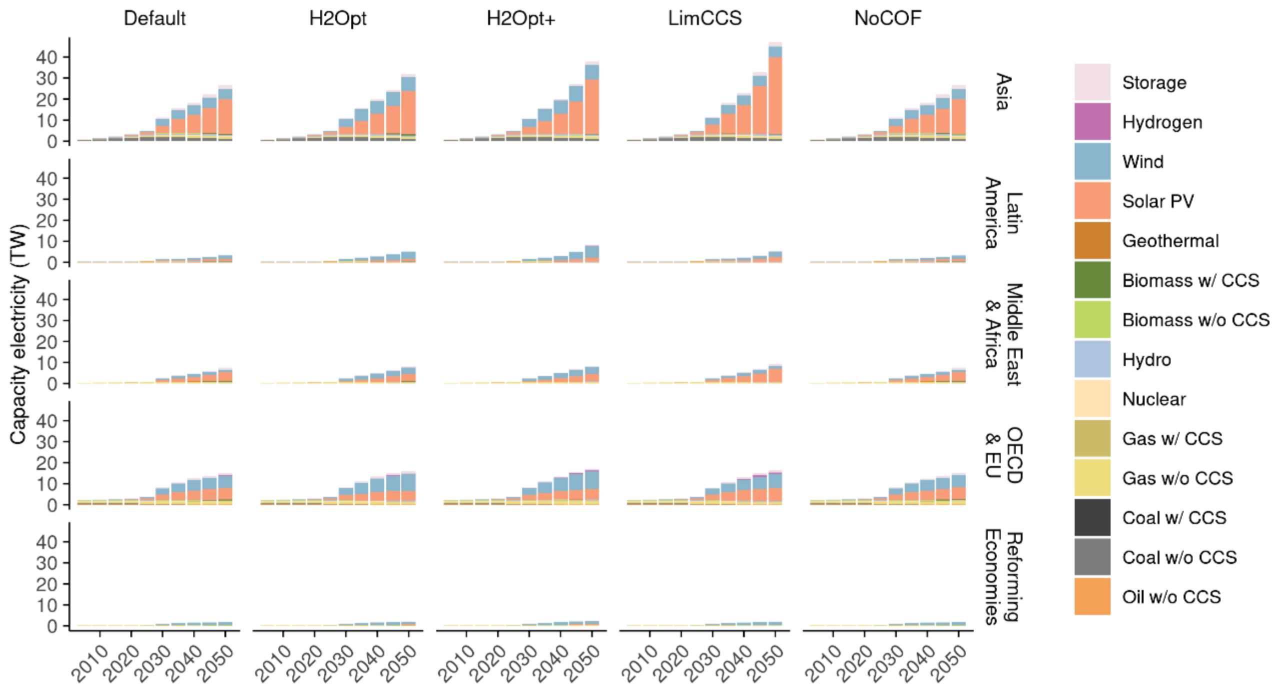

Supplementary Fig. 18. Capacity of electricity by region in the 500 Gt-CO_2 budget scenario.

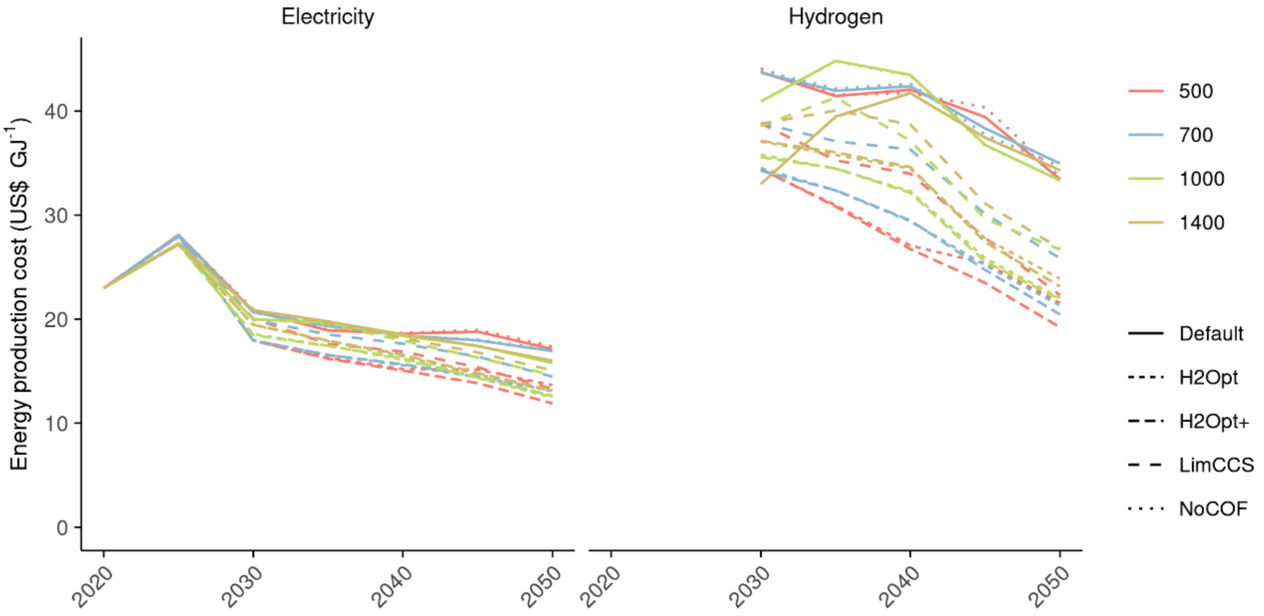

Supplementary Fig. 19. Average production cost comparison between electricity and hydrogen.

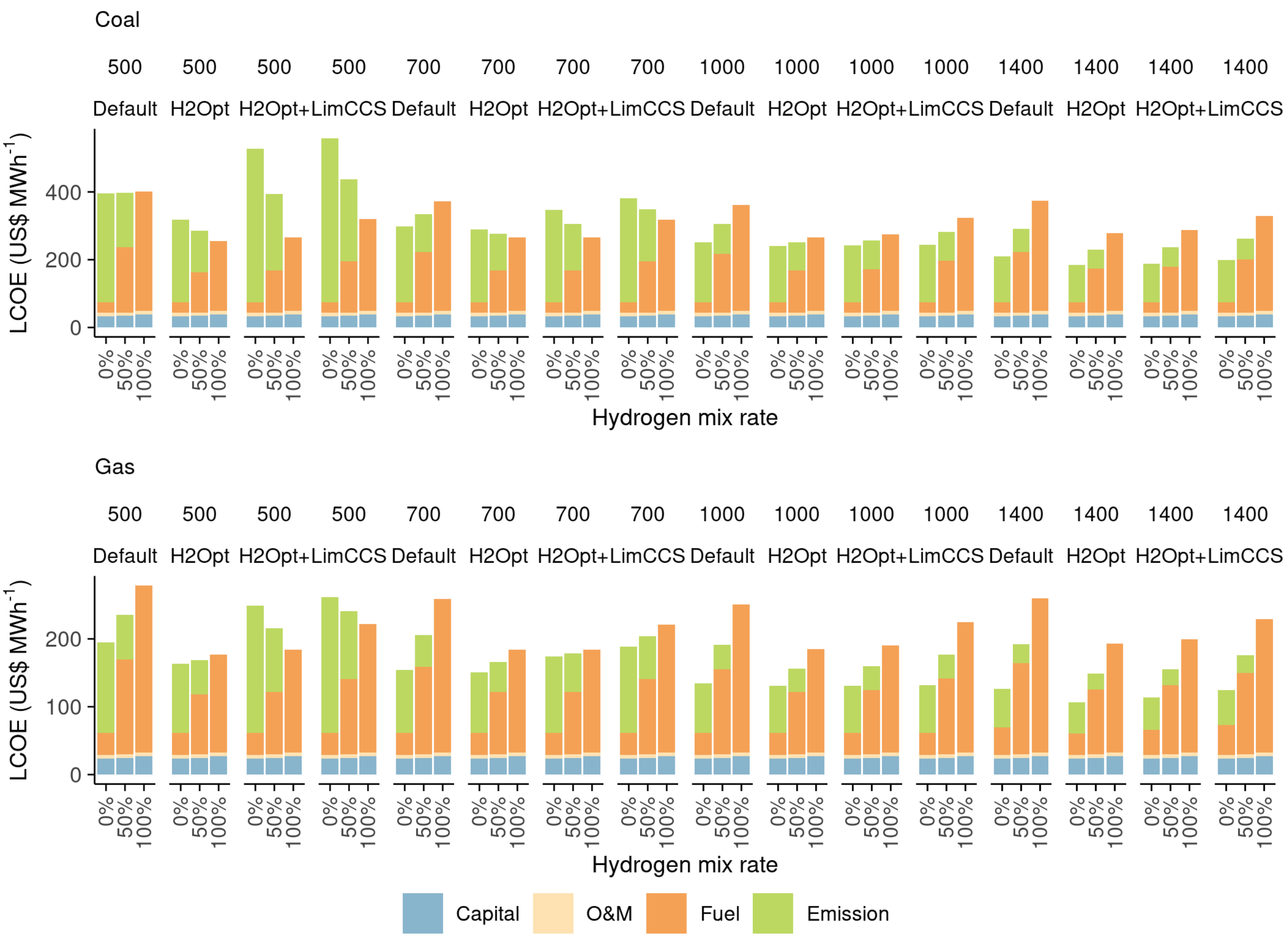

Supplementary Fig. 20. Levelized cost of electricity (LCOE) decomposition of hydrogen co-fired generators in 2050 across all scenarios.

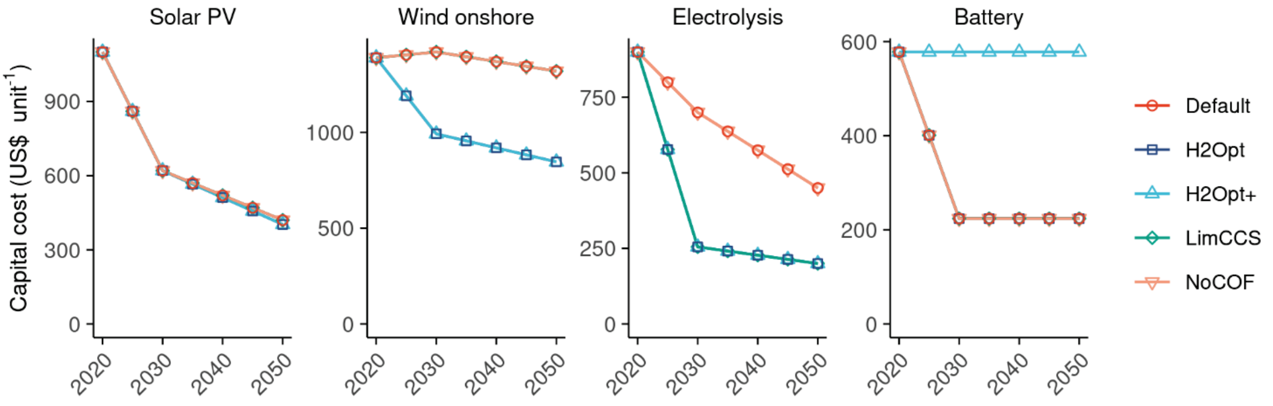

Supplementary Fig. 21. Cost assumptions of technologies associated with hydrogen production. Unit of battery storage and of other technologies is presented as US$ per kWh and US$ per kW, respectively.

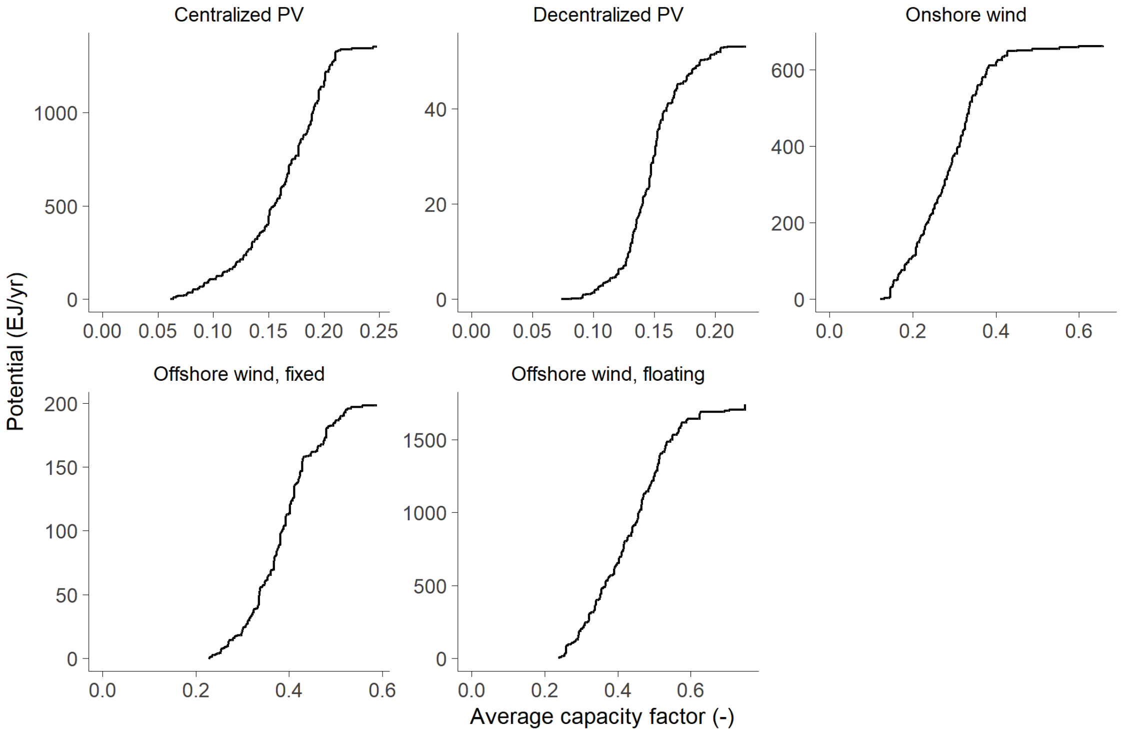

Supplementary Fig. 22. Potential for wind and solar power as a function of average capacity factor.

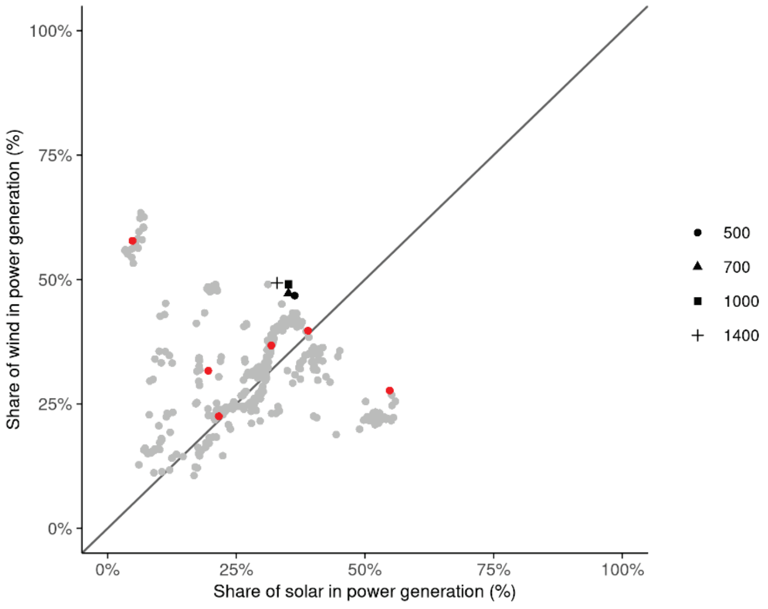

Supplementary Fig. 23 Comparison of the solar and wind share in power generation. Black plots represent the present study's scenarios with Default technology assumptions. Grey and red circles represent the IPCC-AR6 scenarios in C1-C3 categories and the Illustrative Mitigation Pathways (IMPs)[1], respectively.

Supplementary Tables

Supplementary Table 1. Incremental capital cost of retrofitting fossil fuel-fired powerplants for hydrogen co-firing [2].

	<50%	50-100%
Retrofit	7%	25%
New construction	3%	15%

Supplementary Table 2. Technological parameter assumptions for PHES and CAES [3,4,5].

	PHES	CAES
Capital cost (US$ kW^{-1})	1013	600
O&M cost (US$ kW^{-1})	8	4
Lifetime (years)	60	30
E/P ratio (hours)	1000	100

Supplementary Table 3. Summary of technological and socio-economic dimensions of scenarios.

Name	Scenario classification	Description
Default	Default	- Model default assumptions
H2Opt	Default	- Optimistic assumptions for hydrogen co-firing
		- Hydrogen electrolyzer cost reduction [6]
		- Solar and wind power cost reduction [7,8]
LimCCS	Default	- CCS and bioenergy limitation [9]
H2Opt+	Default	- Hydrogen electrolyzer cost reduction [6]
		- Solar and wind power cost reduction [7,8]
		- CCS and bioenergy limitation [9]
		- No new construction of seasonal storage (CAES and PHES)
		- No cost reduction of battery storage [10]
NoCOF	Default	- No hydrogen co-firing
		- Other conditions are same with the Default.
H2Opt_SSP1	Sensitivity	- GDP and population assumptions are based on SSP1 [11].
		- Other conditions are same with the Default.
H2Opt_SSP3	Sensitivity	- GDP and population assumptions are based on SSP3 [11].
		- Other conditions are same with the Default.

Supplementary Notes

Supplementary Note 1. Representation of the technology retrofit process in the energy system model

$$VS_{r,i,l} = VR_{r,i,l} + \sum_{y} VSC_{r,i,l,y} \tag{1}$$

$$VSC_{r,i,l,h} = ss_{r,i,l,h} + \sum_{l_1} VC_{r,i,l_1,l,h} - \sum_{l_1} VC_{r,i,l,l_1,h} \tag{2}$$

$$VTC = \sum_{r,i,l}\left(VR_{r,i,l} \cdot c^{n}_{r,i,l} + VX_{r,i,l} \cdot g^{o}_{r,i,l}\right) + \sum_{r,i,l,l_1,h}\left(VC_{r,i,l,l_1,h} \cdot c^{r}_{r,i,l,l_1,h}\right) + \sum_{r,i,k}\left(VE_{r,i,k} \cdot g^{e}_{r,i,k}\right) + \sum_{r,i,m}\left(VQ_{r,i,m} \cdot \zeta_{r,i,m}\right) \rightarrow \min \tag{3}$$

$$c^{n}_{r,i,l} = b^{n}_{r,i,l} \cdot \left(1 - sc^{n}_{r,i,l}\right) \cdot \frac{\alpha_{r,i,l} \cdot \left(1 + \alpha_{r,i,l}\right)^{t_{r,i,l}}}{\left(1 + \alpha_{r,i,l}\right)^{t_{r,i,l}} - 1} \tag{4}$$

$$c^{r}_{r,i,l,l_1,h} = b^{r}_{r,i,l,l_1} \cdot \left(1 - sc^{r}_{r,i,l,l_1}\right) \cdot \frac{\alpha_{r,i,l_1} \cdot \left(1 + \alpha_{r,i,l_1}\right)^{t^{r}_{r,i,l,h}}}{\left(1 + \alpha_{r,i,l_1}\right)^{t^{r}_{r,i,l_1}} - 1} \tag{5}$$

$VC_{r,i,l,l_1,h}$	Retrofit of existing technology l, which is introduced in year h with new one l_1.
$VSC_{r,i,l,h}$	Amount of existing technology introduced in year h.
$VR_{r,i,l}$	New installation of technology l.
$VX_{r,i,l}$	Technology stock in operation.
$VE_{r,i,k}$	Energy inputs.
$VQ_{r,i,m}$	Emissions.
$ss_{r,i,l,h}$	Amount of existing technology introduced in year prior to year h.
$c^{n}_{r,i,l}$	Annualized capital cost of new construction.
$c^{r}_{r,i,l,l_1,h}$	Annualized additional cost of technology retrofit.
$b^{n}_{r,i,l}$	Capital cost.
b^{r}_{r,i,l,l_1}	Additional cost of technology retrofit.
$g^{o}_{r,i,l}$	Annual fixed operation and management cost.
$g^{e}_{r,i,k}$	Annual energy cost.
$\zeta_{r,i,m}$	Emissions cost.
VTC	Total energy system cost

Supplementary Note 2. Wind and solar potential estimation

Wind and solar potentials were estimated by analyzing climate, weather, and land information within 0.5° x 0.5° grid cells (see Supplementary Fig. 22) [12]. Solar irradiance and wind speed data for climate condition were sourced from the Modern-Era Retrospective Analysis for Research and Applications Version 2 (MERRA-2) dataset in 2010 [13, 14]. The climate data was converted to hourly power output and physical potential for solar and wind power following the formulations and parameter settings outlined by Staffel et al. and Pfenninger et al. [15, 16]. We also considered suitable land areas for the estimation of solar and wind potentials. Low energy density areas were excluded from the overall potential estimation, where the annual average wind speeds were less than 4.5 m/s and 5.5 m/s for onshore and offshore wind, respectively. Additionally, areas with an annual average capacity factor below 5% for solar PV were excluded. Solar and wind potentials were categorized into eight grades in each region based on the level of capacity factor.

For wind power and centralized solar PV, International Geosphere-Biosphere Programme (IGBP) land cover data were utilized to identify suitable areas and technical potential [17]. Suitable areas for solar and onshore wind power generation were calculated using a land suitability factor derived from relevant literature [18, 19]. The suitability factor for offshore wind assumed 5%, 40%, and 80% of the ocean area for the respective ranges of 0-10km, 10-50 km, and 50-200 km from the shore [19]. Assumptions for wind turbine capacity density and solar PV panel efficiency were set at 7 MW/km^2 and 16%, respectively. Exclusions were made for non-exclusive economic zone (EEZ) areas for offshore wind areas beyond 200 km from the shore. Additionally, regions with a bathymetry exceeding 50m were designated as floating offshore wind areas. Both solar and onshore/offshore wind potentials excluded protected areas, categorized under IUCN classification I-VI, based on the World Database on Protected Areas (WDPA) [20]. For decentralized solar PV, potential in residential areas was estimated based on population and household consumption [21], utilizing AIM/Hub and AIM/DS data [22, 23]. Potential in non-residential areas was estimated based on area per capita as per Deng et al. [19].

Supplementary References

1. Byers E*, et al.* AR6 Scenarios Database hosted by IIASA. (ed International Institute for Applied Systems Analysis). International Institute for Applied Systems Analysis, (2022).
2. Öberg S, Odenberger M, Johnsson F. Exploring the competitiveness of hydrogen-fueled gas turbines in future energy systems. *International Journal of Hydrogen Energy* **47**, 624-644 (2022).
3. IEA-ETSAP. IEA-ETSAP Technology brief. (2011).
4. Stocks M, Stocks R, Lu B, Cheng C, Blakers A. Global Atlas of Closed-Loop Pumped Hydro Energy Storage. *Joule* **5**, 270-284 (2021).
5. Aghahosseini A, Breyer C. Assessment of geological resource potential for compressed air energy storage in global electricity supply. *Energy Conversion and Management* **169**, 161-173 (2018).
6. IEA. The Future of Hydrogen Seizing today's opportunities. IEA (2019).
7. IRENA. *Future of Solar Photovoltaic: Deployment, investment, technology, grid integration and socio-economic aspects*. International Renewable Energy Agency (2019).
8. IRENA. *Future of wind: Deployment, investment, technology, grid integration and socio-economic aspects*. International Renewable Energy Agency (2019).
9. Luderer G*, et al.* Impact of declining renewable energy costs on electrification in low-emission scenarios. *Nature Energy* **7**, 32-42 (2022).
10. IRENA. *Electricity Storage and Renewables: Costs and Markets to 2030*. International Renewable Energy Agency (2017).
11. Riahi K*, et al.* The Shared Socioeconomic Pathways and their energy, land use, and greenhouse gas emissions implications: An overview. *Global Environmental Change* **42**, 153-168 (2017).
12. Oshiro K, Fujimori S. Role of hydrogen-based energy carriers as an alternative option to reduce residual emissions associated with mid-century decarbonization goals. *Applied Energy* **313**, 118803 (2022).
13. Global Modeling and Assimilation Office (GMAO). MERRA-2 tavg1_2d_rad_Nx: 2d,1-Hourly,Time-Averaged,Single-Level,Assimilation,Radiation Diagnostics V5.12.4. Goddard Earth Sciences Data and Information Services Center (GES DISC) (2015).
14. Global Modeling and Assimilation Office (GMAO). MERRA-2 tavg1_2d_slv_Nx: 2d,1-Hourly,Time-Averaged,Single-Level,Assimilation,Single-Level Diagnostics V5.12.4. Goddard Earth Sciences Data and Information Services Center (GES DISC) (2015).
15. Staffell I, Pfenninger S. Using bias-corrected reanalysis to simulate current and future wind power output. *Energy* **114**, 1224-1239 (2016).
16. Pfenninger S, Staffell I. Long-term patterns of European PV output using 30 years of validated hourly reanalysis and satellite data. *Energy* **114**, 1251-1265 (2016).

17. Friedl MA, Sulla-Menashe D. MCD12C1 MODIS/Terra+Aqua Land Cover Type Yearly L3 Global 0.05Deg CMG V006. NASA EOSDIS Land Processes DAAC (2015).
18. Hoogwijk M. On the global and regional potential of renewable energy sources. Utrecht University (2004).
19. Deng YY*, et al.* Quantifying a realistic, worldwide wind and solar electricity supply. *Global Environmental Change* **31**, 239-252 (2015).
20. UNEP-WCMC, IUCN. The World Database on Protected Areas (WDPA)/OECM Database. UNEP-WCMC and IUCN (2019).
21. Gernaat DEHJ, de Boer H-S, Dammeier LC, van Vuuren DP. The role of residential rooftop photovoltaic in long-term energy and climate scenarios. *Applied Energy* **279**, 115705 (2020).
22. Fujimori S*, et al.* SSP3: AIM implementation of Shared Socioeconomic Pathways. *Global Environmental Change* **42**, 268-283 (2017).
23. Fujimori S, Hasegawa T, Ito A, Takahashi K, Masui T. Gridded emissions and land-use data for 2005–2100 under diverse socioeconomic and climate mitigation scenarios. *Scientific Data* **5**, 180210 (2018).
